# What’s in a pun? Assessing the relationship between phonological distance and perceived funniness of punning jokes

**DOI:** 10.1515/humor-2024-0060

**Published:** 2025-08-26

**Authors:** Anna Palmann, Tristan Miller

**Affiliations:** Institute for Logic, Language and Computation, 1234 University of Amsterdam, Science Park 900, 1098 XH, Amsterdam, Netherlands; Department of Computer Science, University of Manitoba, Winnipeg, MB, R3T 2N2, Canada

**Keywords:** puns, paronomasia, humor perception, phonological distance

## Abstract

Punning is a form of humorous wordplay based on semantic ambiguity between two phonologically similar words – the pun and the target – in a context where both meanings are more or less acceptable. While the pun is expressed explicitly, the target is invoked implicitly in the text. Previous work has attempted to quantify and compare phonological features of puns and their targets, looking at correlations with the understandability of the jokes in which they occur. Our study quantifies the phonological distance between pun and target words and assesses possible correlations with funniness ratings of the corresponding jokes. Our statistical analyses on a large dataset of puns reveal a significant negative correlation between phonological distance and perceived funniness for two of the four phonological distance measures we applied. This finding supports the hypothesis, often (implicitly) made in previous research but never verified at this scale, that lower phonological distance between a pun and its target is associated with higher funniness ratings. The parameters of our study suggest that future work should examine the semantic features of pun and target in order to create a more holistic understanding of what contributes to the perceived funniness of punning jokes.

## 1 Introduction

Punning is a form of humorous wordplay based on semantic ambiguity between two words that are phonologically similar or identical, in a syntactic context in which both meanings are more or less acceptable (Hempelmann and Miller 2017). Humor in punning jokes relies strongly on phonological features of both the pun word, which is explicitly expressed in the joke, and the target word, whose meaning is invoked implicitly. Several attempts have been made to quantify those features and investigate possible correlations with the comprehensibility of a punning joke (for an overview of which see Hempelmann and Miller 2017). However, to date there has been no empirical investigation of the relationship of phonological features between puns and their targets and the perceived funniness of the joke that contains them. The study described in this article investigates this question, in particular through analyses of correlations between funniness ratings1Carrell (1997) distinguishes between the constructs of joke competence and humor competence: the former refers to one’s ability to determine whether or not a given text is a joke, and the latter to the capacity to appreciate, to some degree, the humor in a text that has already been recognized as a joke. In this paper, we use the term funniness in the context of humor competence, referring to a judgement as to whether one text is perceived as funnier or more amusing than another, assuming that the recognition of the texts as jokes is a given. of punning jokes obtained in a crowdsourcing study and various phonological distance measures of the respective pun–target pairs.

From previous analyses of puns (e.g., Lagerquist 1980), we expect punning jokes with lower phonological distance between the pun and target word to be associated with higher funniness ratings. More simply put, the more the pun and its target sound alike, the funnier the punning joke should be perceived as. Consequently, we would expect homophonic puns – those in which the pun and target sound exactly the same – to be perceived as funnier than heterophonic ones, in which the pun and target are further apart in sound. The statistical analyses we present in this paper provide empirical evidence for this tendency. As we discuss in later sections, however, homophony alone cannot be the sole determinant of the degree of funniness of a punning joke. Identifying and measuring the contributions of the pun’s semantic features, the joke’s discourse context, and the cultural background and personal humor preferences of the audience would be promising avenues for future research.

## 2 Background

In incongruity-resolution theories, humor is viewed as two-step process (Uekermann et al. 2007). In the first step, an incongruous element is detected among several incompatible elements, and in the second, this element is linked in a sense-making manner to the context, leading to resolution of the incongruity and a subsequent humorous sensation. Under this interpretation, incongruity is a necessary feature and its resolution a sufficient feature of humor. According to Hempelmann (2003), the questions of whether an element is incongruous in a certain context and whether there is script opposition present is a matter of situation, cultural context, and individual knowledge resources. All of these can vary greatly and influence incongruity to different extents.

Like many others, Attardo et al. (1994) acknowledge phonological similarity to be crucial for incongruity resolution in puns, and use phonological distance as a core measure for the sound-related features in punning jokes. Recognizing that the pun and target word in a punning joke can be similar in sound to varying degrees, Attardo et al. (1994) posit the existence of a threshold in phonological distance that, when reached, makes it impossible to understand a joke. This idea goes at least as far back as Hausmann (1974), who investigated French punning jokes based on the number of differing phonemes between pun and target word and found the largest possible distance to be four phonemes.

Lagerquist (1980) investigated the types of phonemic changes that occur between pun and target words in English puns, among them insertions (additions of phonemes), deletions (omissions of phonemes), mutations (alterations of phonemes), and transpositions (changes in the position of phonemes). Most of the changes were found to involve consonants, and most mutations were focused on a single feature. Since only a quarter of her data showed alterations in the initial segment of the word, she hypothesized a special role for the preservation of the beginning of the sound sequence in the perception of homophony. Other significant observations were the tendencies to preserve the number and stress pattern of syllables between the pun and target. These findings indicate that in the production of punning jokes, the speaker aims to preserve homophony. Keeping in mind the goal of facilitating target recovery, it seems reasonable that the applied changes in the phoneme structure are not major.

In his book on the phonology of puns, Sobkowiak (1991) quantified pun–target sound similarity based on distinctive features and was able to confirm the hypothesis made by Lagerquist (1980) that consonants are more likely than vowels to undergo changes or deletions. He further noted that the understandability of a punning joke increases when the consonant structure is kept intact, since vowels are more mutable and carry less information.

Also in line with previous research, Fleischhacker (2005) states that the pun word needs to be sufficiently similar to the target word for the latter to be recovered. Further, the degree of common representation within a corpus (determined by calculating the ratio of the frequency of a pun–target combination observed in the corpus to the frequency that would be expected if pun types occurred at random, based on the number of relevant word pairs in English) is positively correlated with whether a punning joke is recognized as such in a conversational setting and therefore considered successful. However, according to Hempelmann (2003), while pun–target similarity can explain a higher rate of target recovery, it cannot explain greater funniness. Fleischhacker (2005), on the other hand, assumes that “good” punning jokes with a subtle but quickly recognizable phonological relationship between pun and target are also those that are perceived as funnier.

Jaech et al. (2016) present a study similar to the one described in the present paper. They design a computational model for the recovery of the target word in a punning joke based on the position of the pun word within a sentence context. Rather than calculating phonological similarity between pun and target, they model the transformation probability of the two by averaging the log-likelihood value from their model over the phonemes in the pun, resulting in a phonetic-edit score for each pun–target pair. They use this model to determine whether there is a relationship between the funniness of a punning joke, which they assess through participant ratings, and the phonological similarity of its pun–target pair. Although their statistical analysis finds evidence for such a relationship, their dataset contains only seventeen punning jokes, which calls into question how well their results generalize. A further point of concern is that their funniness ratings were applied with a Likert scale, a practice that has been criticised in the context of humor studies by (inter alia) Shahaf et al. (2015) and Simpson et al. (2019).

Building in part on the notions of joke competence and humor competence (Carrell 1997), Hay (2001) defines a progression of processes associated with the experience of humor – to wit, recognizing, understanding, appreciating, and agreeing with a humorous statement. These processes build upon each other, so that recognition and understanding are necessary for appreciation. Target recovery in punning jokes connects to recognition and understanding in this framework, but it does not necessarily entail appreciation. Our study, which focusses on the perceived funniness of jokes, is connected to the appreciation process, although as Hay (2001) indicates, appreciation is not always possible to disentangle from agreement.

## 3 Methods

Our study builds on a previously published dataset of punning jokes annotated with information concerning the pun and target and with pairwise funniness judgements. We added further annotations in the form of phonemic transcriptions of puns and targets and used these to calculate four different phonological distance measures between the puns and targets. We then performed correlation analyses between the various phonological distance measures and the funniness ratings. The following two subsections provide further details on the data and distance measures.

### 3.1 Dataset

The dataset for this study is based on the one originally created for the SemEval-2017 evaluation campaign on the computational detection and interpretation of puns (Miller et al. 2017) and later extended by Simpson et al. (2019) for a study on funniness prediction. The original SemEval-2017 dataset contains 4030 humorous and non-humorous short texts sourced from professional humorists and online collections of jokes and aphorisms. Each of the punning jokes in the dataset contains a single pun involving a single pair of content words (i.e., nouns, verbs, adjectives, or adverbs) and is annotated as being either homographic or heterographic (i.e., the pun and target word have the same or different spellings, respectively).

Simpson et al. (2019) applied funniness annotations to these texts using pairwise human judgements where participants were asked to select the funnier of two punning jokes presented to them simultaneously. This technique keeps the annotators’ cognitive burden low and, unlike Likert scale ratings, is not affected by biases towards high, low, or middle values or changes in individual rating behavior over time. A numeric score for each instance can be mathematically inferred from the pairwise judgements using a random utility model, as in Gaussian process preference learning, or GPPL (Chu and Ghahramani 2005). Gaussian processes are distributions over functions of input features, where relationships and covariances between instances can be computed based on the posterior distribution, which is estimated over the utilities of an instance given a set of pairwise labels and their features. In contrast to MaxDiff-based approaches to pairwise judgements (e.g., best–worst scaling), GPPL applies machine learning techniques that make it particularly robust when working with noisy or small data (Simpson and Gurevych 2018).

To create their augmented dataset, Simpson et al. (2019) assigned the SemEval-2017 texts randomly into 28,210 pairs and presented these to 1,063 participants recruited through the Amazon Mechanical Turk crowdsourcing platform. All participants accessed the platform from the US and self-identified as native English speakers, which was the sole inclusion criterion. Each participant annotated anywhere from 10 to 2,200 pairs according to which text (if either) they considered to be funnier. After verifying that inter-annotator agreement was high, Simpson et al. (2019) applied GPPL to derive numeric funniness scores for the 4030 individual texts, which we normalized to range from 0 to 1.

Our study initially selected the 2,772 texts from this dataset that the original compilers annotated as punning jokes (as opposed to non-humorous texts, or joke texts whose humor relies on a mechanism other than punning). We applied ARPAbet phonemic transcriptions (Shoup 1980) to the pun and target words of each text automatically using the CMU Lexicon Generation Tool2http://www.speech.cs.cmu.edu/tools/lextool.html. and then manually checked and post-corrected the output. We then programmatically converted the ARPAbet transcriptions to Unicode International Phonetic Alphabet (IPA) symbols for later use with the software library (see below) that we use to calculate phonological distance. Incompatibilities between the two systems obliged us to discard some of the texts, leaving us with a total of 2,718 punning jokes, of which 1,131 Miller et al. (2017) had originally annotated as heterographic and 1,587 as homographic.

### 3.2 Linguistic distance measures

Linguistic distance is defined as the amount of distinctness between words, and is often characterized by a difference in sound. Over the past several decades, several computerized measures have been developed to measure phonological differences between words (Sanders and Chin 2009). We use the Python library Abydos (Little 2020), which implements various string-based distance measures and metric classes that operate on individual IPA characters or sequences thereof. Many of these measures account for phonetic features associated with the character, such as (for consonants) place and manner of articulation, syllabicness, voicedness, nasality, laterality, retroflex quality, and aspiration.3We discovered that Abydos does not recognize the whole range of IPA characters; one phoneme (the open-mid back unrounded vowel “ʌ”) was not initially defined. In this case, we adapted the source code to include this phoneme by defining the corresponding features. We selected the following four distance measures for our study, as summarized in Table 1:

**Table 1:** Overview of phonological distance measures.

Type	Costs based on	Phonetic features used
Levenshtein	indel, substitution	none (characters)
Covington	indel, substitution	none (consonants, vowels, glides)
ALINE	indel, substitution, expansion, contraction	weighted, multivalued
Phonetic edit distance	indel, substitution, transposition	weighted, 3-valued (absent, present, neutral)

Levenshtein distance (Levenshtein 1966) is a measure of edit distance based on the number of operations needed to convert one string of characters into another string. It assigns a numerical cost for each edit operation (unit insertion, unit deletion, unit substitution), with the sum of costs representing the total distance. Distance is therefore based solely on the positional relationship of the characters to each other, without accounting for phonological features. This makes it widely applicable but less linguistically informative.

Covington distance (Covington 1996) is a distance function based on consonants, vowels, and glides but not their finer phonological features. To calculate the distance between characters, it uses an evaluation metric consisting of an 8-tuple of alignment costs for insertions, deletions, and substitutions. The value of the distance between two characters ranges from 0 to 100, where 0 means identity and 100 maximal difference.

ALINE distance (Kondrak 2002) calculates the relationship of characters based on their phonetic features as defined in the IPA, along with a set of feature salience weights. These features are not binary but multi-valued, which results in around twenty distinct features including place of articulation, voice, roundness, etc. The weighted features are used to calculate a similarity score for insertion, deletion, substitution, expansion, or contraction, with additional costs for vowel substitution, expansion, or contraction.

Phonetic edit distance (Little 2020) is a custom calculation created by the developer of Abydos. A variation of Levenshtein distance adapted for strings in IPA, it compares individual phonemes based on their feature similarity. The cost function is again based on insertions, deletions, substitutions, and transpositions, with the latter having a lower cost value than insertions and deletions (indels) or substitutions. It also allows for feature weighting, but unlike ALINE it does not use multiple values per feature.

We arrived at this selection of measures for a variety of reasons. Levenshtein distance was chosen as it is one of the simplest, most generic, and by far the most widely used edit distance measures; it provides a nearly language- and linguistically agnostic view of our task. We chose Covington distance as one of the simplest possible linguistically informed distance measures; it encodes marginally more knowledge than Levenshtein by accounting for basic differences between speech sounds. With their fine-grained representation of phonetic features, ALINE and Phonetic edit distance represent the most detailed approaches available in Abydos. ALINE in particular has seen widespread use in (computational) linguistic studies, and seemed especially promising to us for its use of weighted multi-valued features that allow for the investigation of more fine-grained phonological differences.

In addition to the four phonological distance measures, whose values we normalize to the range 0 (maximal dissimilarity) to 1 (complete identity), we also computed correlations between the homographic and heterographic subsets of the data, as originally annotated by Miller et al. (2017). As homographic puns are generally also homophonic, while heterographic puns are generally also heterophonic, taking the dataset’s pre-existing division between homographic and heterographic puns and using it as proxy for homophony and heterophony provides us with an expedient yet reasonably accurate coarse-grained (indeed, binary) measure of phonological distance.

## 4 Results

**Figure 1:**
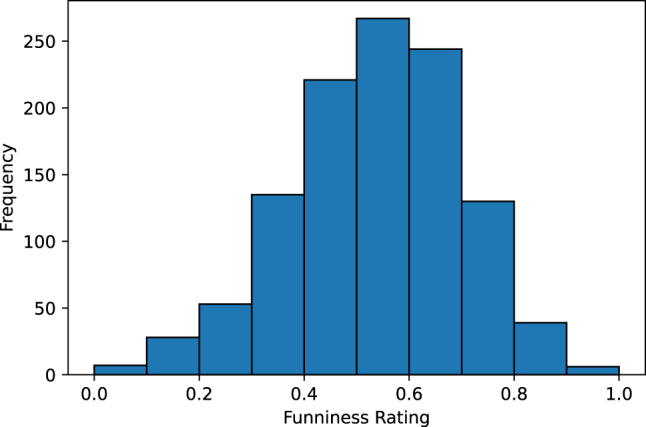
Frequency distribution of funniness ratings (normalized and quantized).

Figure 1 shows the frequency distribution of normalized, quantized funniness ratings calculated using GPPL across the entire dataset. Most texts scored around 0.6, which is slightly above the median. This normal distribution indicates that most punning jokes were rated in a medium range for funniness with only few of them rated as very low or very high in funniness.

**Figure 2:**
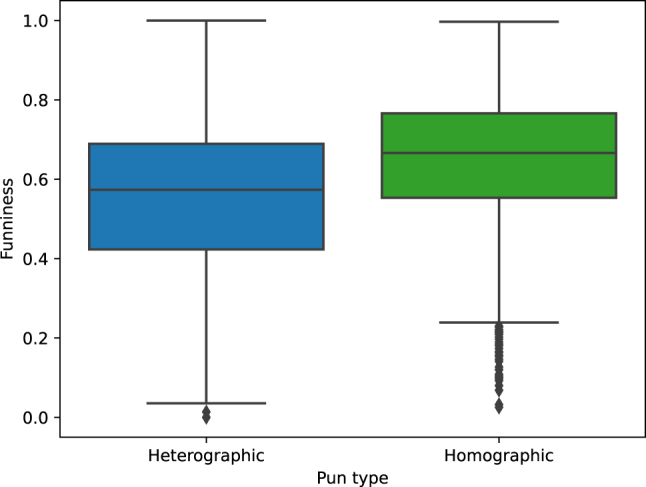
Funniness ratings for heterographic versus homographic puns.

Our correlation analysis begins with a group comparison between the homographic and heterographic puns. We performed an independent samples *t*-test comparing mean funniness ratings of the two subsets and found a significant difference in the scores for homographic ( $\bar{x}$ = 0.60, *σ* = 0.14) and heterographic ( $\bar{x}$ = 0.52, *σ* = 0.16) puns (*t*(2,770) = 13.45, *p* < 0.0001). The box plots in Figure 2 show a direct comparison between the funniness ratings of the two pun types, indicating that funniness ratings for the homographic puns are higher than those of the heterographic ones. What is striking here is the large variance in funniness ratings in both directions, indicated by the relatively large whiskers for both classes. Further, there seems to be a number of outliers in the lower range of funniness ratings, especially for the homographic group.

**Figure 3:**
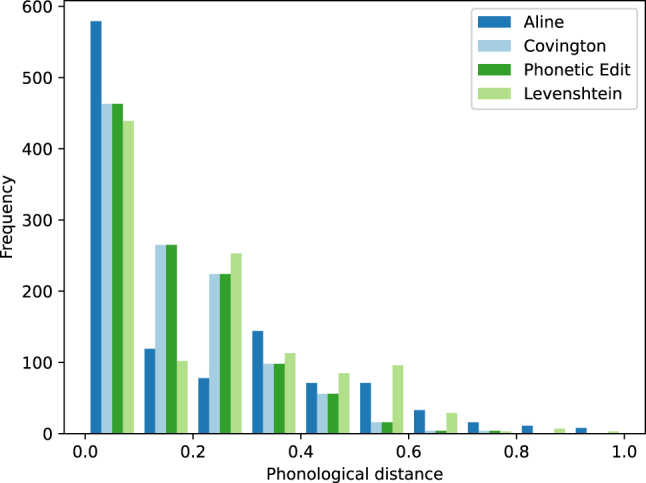
Comparison of phonological distance measures (normalized and quantized).

Next, we compare phonological distance measures for the four real-valued distance metrics. Figure 3 shows the frequency distribution of the different phonological distance measures after normalizing and quantizing them. As can be seen in the plot, the majority of puns under investigation are characterized by a low phonological distance between pun and target word. We computed Spearman’s rank correlation to assess the relationship between all four phonological distance measures. There was a positive correlation between ALINE and Levenshtein distance (*ρ* = 0.91, *p* = 0.0), ALINE and Covington distance (*ρ* = 0.83, *p* < 0.0001), ALINE and Phonetic edit distance (*ρ* = 0.83, *p* < 0.0001), Levenshtein and Covington distance (*ρ* = 0.94, *p* = 0.0), Levenshtein and Phonetic edit distance (*ρ* = 0.94, *p* < 0.0001), and Covington and Phonetic edit distance (*ρ* = 1.0, *p* < 0.0001).

We now present separate statistical analyses for each distance measure in order to identify possible correlations with the funniness ratings. For this, we once again use Spearman’s rank correlation, finding a significant negative correlation for Levenshtein distance (*ρ* = −0.089, *p* = 0.021) as well as for ALINE distance (*ρ* = −0.165, *p* = 0.0003). This indicates that lower phonological distance values are associated with higher funniness ratings. For the other phonological distance measures, we observed no significant correlation with the funniness ratings.

**Figure 4:**
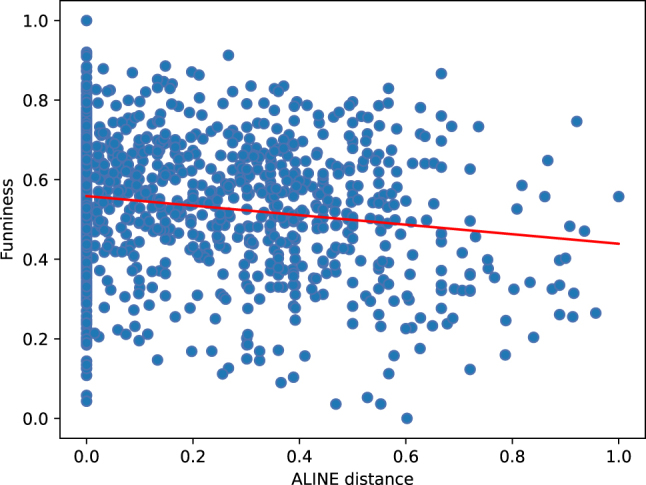
Negative correlation between ALINE distance and funniness ratings.

**Figure 5:**
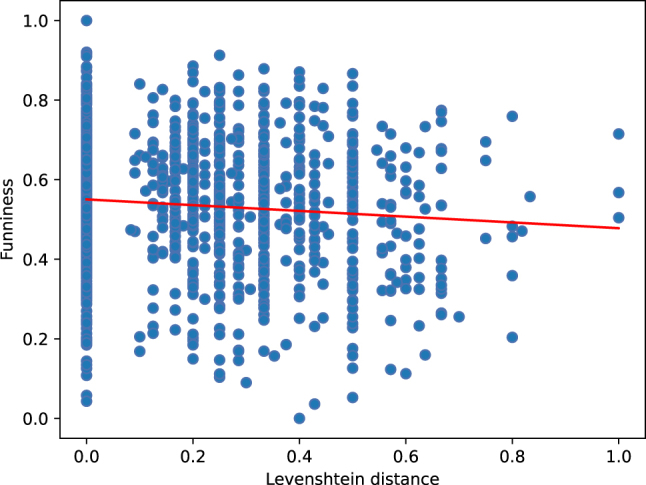
Negative correlation between Levenshtein distance and funniness ratings.

Figures 4 and 5 show the respective scatter plots for the negative correlations (indicated by the regression line) between ALINE distance and GPPL funniness ratings, as well as between Levenshtein distance and GPPL funniness ratings.

## 5 Discussion

Our empirical findings are in line with Fleischhacker’s (2005) oft-repeated assumption that punning jokes in which the pun and target are phonologically closer are perceived to be funnier. In the broader framework of linguistic humor theory, this can be at least partially explained through the concept of target recovery. In particular, theories on the linguistic mechanisms involved in pun processing hold that the implied target word must be (effortfully) recovered by the listener in order for them to understand the punning joke and perceive it as humorous. When the pun and target are closer in sound, it can be easier for the listener to activate the second meaning because there is no need for phonological “stretching.” What remains for the listener is a cognitive exercise on the semantic level in order to evoke the second meaning. Of course, this also holds true for the homographic versus heterographic case, so that target recovery is easier in homographic rather than heterographic puns – not necessarily because of their phonological features but simply also because of the identical orthography of pun and target word.

When comparing mean funniness ratings for homographic and heterographic puns, we found a large variance in both pun types. This raises the question of how meaningful this binary feature is. The fact that raters perceived the funniness of the individual punning jokes very differently might indicate that how funny a larger audience finds a joke cannot be easily measured on a numerical scale – not even when using more reliable and robust approaches that derive numerical ratings from pairwise comparisons.

Our assessment of correlations between the individual phonological distance measures and perceived funniness revealed significant negative correlations for the ALINE and Levenshtein distance measures. Of all metrics we applied, Levenshtein is the most basic; it focusses exclusively on the relative positions of characters without accounting for the phonological information they encode. Its negative correlation with funniness ratings suggests that target recovery may not always involve particularly sophisticated processing. Alternatively, this correlation could be related to the fact that the punning jokes were presented in writing, leading the raters to focus on orthographic rather than phonological or acoustic differences. In contrast to Levenshtein, ALINE distance accounts for specific phonetic features in a fairly sophisticated way. The significant negative correlation with funniness ratings that we observed points to a role in funniness perception beyond mere orthographic similarity, even when the stimuli are presented in writing.

A correlation analysis among our four distance metrics indicated a positive correlation between all of them. This raises the question why only ALINE and Levenshtein distance, but not Covington and the Phonetic edit distance, are significantly correlated with funniness ratings. We suppose that, in the case of Covington, the coarse-grained differentiation of characters into consonants, vowels, and glides is simply not meaningful for accurately judging pun–target similarity, or at least that it obscures the distinctions necessary to make such judgements. With respect to Abydos’s Phonetic edit distance, this measure uses feature weights that are distinct from those derived and explained by Kondrak (2000) for ALINE, and whose provenance is not recorded in the library’s official documentation. Further details on the origin and intended purposes of these feature weights could help explain the null results we observed.

### 5.1 Limitations and further directions

Several factors might have limited this investigation and influenced its results. Regarding the situational context, all of the puns used in our study are self-contained jokes in which humor does not directly relate to the context but more on general knowledge (Dynel 2010; Ritchie 2005). Since there is no wider conversational or discourse context to account for, no differentiation based on the relationship of pun word and context as proposed by Hempelmann (2003) can be made. Additionally, the rather mechanical way in which Simpson et al. (2019) sourced the funniness judgements we used – namely, by presenting jokes to annotators in series, thus priming them for humorous stimuli – is not fully translatable to a spontaneously emerging humorous situation. The annotators were not required to actively enter a state of playful, humorous conversation (Raskin 1985) during joke processing, nor to distinguish between their appreciation of and agreement with the jokes (Hay 2001), which may have affected the reliability of the data.

Another issue arising from the data collection process used by Simpson et al. (2019) is the lack of demographic information on the raters. The only requirements for raters to take part in their annotation study were to be located in the US and to self-identify as a native English speaker. Participants were not asked to give any further demographic information, such as their age, gender, or country of origin, nor were they given any tests or questionnaires to determine their personal preferences regarding humor, their current mood, or their proficiency in English or other languages. This low threshold for participation facilitated the collection of large amounts of quality-controlled data appropriate for the purpose of the original study, but at the same time means that this data is of limited utility for answering other research questions. Given sufficient resources to replicate Simpson et al.’s (2019) annotation study with access to more personal information on the participants, future studies could assess possible correlations between funniness ratings and rater- or population-specific factors, perhaps also accounting for Hay’s (2001) distinction between humor appreciation and agreement. An alternative, more qualitative but less scalable approach for finding factors that play a role in funniness ratings would be to ask participants directly to justify their individual funniness ratings.

Another limitation regarding design of our study is the way in which phonological features are encoded in the metrics used for the calculation of phonological distance. A general problem with feature-based metrics is that sounds perceived as similar by human raters often still differ in a disproportionately large amount of features. Thus, human raters may capture subtle differences between phonological features in a different way than feature-based metrics do. A major reason for this seems to be that standard distinctive features are based on articulation rather than acoustics, and thus focus on the creation rather than the perception of a sound. Hempelmann (2003) stresses that it is not only phonological features but also acoustic ones that play a role in the perception of phonological distance. Other phonological treatments of pun–target differences (surveyed in Hempelmann and Miller 2017) suggest they are further constrained by syllable stress, phonological markedness, and the position of changed features within a syllable or word; none of these are explicitly accounted for by the off-the-shelf metrics we employed.

Although a future study on pun–target comparison could investigate the use of acoustic measures, or more sophisticated phonological measures customized for the criteria at play in punning, use of the Simpson et al. (2019) funniness ratings may still be problematic since they were obtained from written stimuli, and generally speaking covered no puns that cross word boundaries. This may have made the differences between homographic and heterographic puns more pronounced as compared to an experimental setting in which punning jokes are presented in the form of an auditory stimulus. The latter allows for more freedom in acoustic interpretation, whereas differences in spelling necessarily underline the heterographic character of pun and target.

Finally, our study has not taken into account the semantic relationship between the pun and target word. It is reasonable to assume that, apart from phonological distance, the degree of semantic similarity between the two has an impact on the perceived funniness of a punning joke, since this too influences how easily the second meaning is retrieved. Further studies could therefore aim to identify semantic relationships between puns and their target words and to investigate possible correlations with funniness ratings. Such experiments could make use of relationships between entries in semantic databases, such as WordNet or Word2Vec.

## 6 Conclusions

This study aimed to investigate the relationship between the phonological distance between pun and target word on the one hand, and funniness ratings on the other hand. Statistical analyses on a large dataset of punning jokes rated for funniness revealed a negative correlation between phonological distance and funniness, providing empirical evidence that punning jokes where the pun and target are closer in sound are generally perceived as funnier.

In a broader humor-theoretic view, punning is based on linguistic ambiguity and is characterized by a resolution of incongruity during the processing of a joke. Successful recovery of the target word is a prerequisite for this incongruity resolution. Lower phonological distance between pun and target is hypothesized to facilitate target recovery, which may explain the higher funniness ratings we observed for more homophonous puns. While the limitations of our data and experimental setup do not allow for firm conclusions concerning the direct causal influence of phonological distance on the perception of funniness, our results are in line with previous findings and lay the groundwork for more in-depth investigations that account for additional factors.
